# The population-based Tromsø 8 study and e-mental health

**DOI:** 10.1192/j.eurpsy.2024.1134

**Published:** 2024-08-27

**Authors:** R. Wynn, G. Bellika, L. G. Fernandez, V. T. Salcedo

**Affiliations:** ^1^Clinical Medicine, UiT The Arctic University of Norway, Tromsø; ^2^Department of Education, ICT and Learning, Østfold University College, Halden, Norway; ^3^Clinical Medicine, University Miguel Hernandez, Alicante; ^4^ITACA, UPV, Valencia, Spain

## Abstract

**Introduction:**

Ageing populations with increased needs, rising costs of traditional services, and new technologies are some factors driving the use of e-health services. A Norwegian study with data from 2015-2016 found that 13.5% had used apps, 7.3% had used social media, and 5% had used video services for health purposes. Little is known about the effects of many online health services, but in general they seem to increase knowledge and make most people feel reassured, although some users feel more anxious or confused after using such tools. Recent technological developments have resulted in new online health services, including AI-based technologies. More updated knowledge regarding the population’s use of e-health services in general and e-mental health services in particular, is needed.

**Objectives:**

The objective here is to provide information about an upcoming large population-based epidemiological study and how it addresses e-health and e-mental health.

**Methods:**

We introduce the upcoming 8th version of the epidemiological Tromsø Study and discuss its importance to the field of e-mental health.

**Results:**

The Tromsø epidemiological study has since 1974 taken place in the Norwegian municipality of Tromsø. It contains information on a range of topics within health and illness, including topics from many medical specialities, psychiatry and substance use. In the upcoming 8th version of the study (2025-2026), more than 33,000 people aged above 40 will be invited to participate. The main questionnaire will include questions relating to a wide variety of topics, including on e-health use. We suspect the importance of e-health and e-mental health have increased lately, and we will examine how the use of e-health may impact mental health.

**Conclusions:**

Community-based studies, such as the Tromsø Study, allow researchers to study associations between many different variables, including mental health and e-health. The upcoming Tromsø 8 study will enable us to study e-health use and its relationship to mental health in a large sample representative of the Norwegian population.

**Disclosure of Interest:**

None Declared

